# Evaluation of antenatal care quality for preterm birth prevention using an auditable scoring system: A retrospective, descriptive, longitudinal study in Sydney, Australia

**DOI:** 10.18332/ejm/191993

**Published:** 2024-09-24

**Authors:** Hasan Rawashdeh, Rhett Morton, Jon Hyett

**Affiliations:** 1 Jordan University of Science and Technology, Irbid, Jordan; 2 Royal Prince Alfred Hospital, Sydney, Australia; 3 Department of Obstetrics, Gynaecology and Neonatology, The University of Sydney, Sydney, Australia

**Keywords:** Australia, cervical length measurement, quality assurance, premature birth, clinical audit, antenatal care

## Abstract

**INTRODUCTION:**

Preterm birth continues to be one of the most significant contributors to perinatal death. This study aims to evaluate the quality of antenatal care provided to women delivering preterm.

**METHODS:**

This was a retrospective, descriptive, longitudinal review of all women who had antenatal care within a single Australian tertiary hospital and delivered spontaneously between 24 and 37 weeks of gestation, using an auditable scoring system assessing potential interventions for prevention of preterm birth. The review was limited to singleton pregnancies without fetal abnormalities delivering between January 2013 and April 2015. The audit tool was developed by reference to established ‘best practice’ guidance for prediction and prevention of preterm birth based on Royal Australian and New Zealand College of Obstetricians and Gynaecologists guidelines and published literature. Different pathways were assessed for women deemed either low- or high-risk at the outset of antenatal care.

**RESULTS:**

A series of 161 pregnancies that delivered preterm (between 24 and 37 weeks’ gestation) were reviewed. The quality of antenatal care was scored ‘good’ in 42.9% and 50% of high-risk and low-risk women, respectively. Care was scored ‘adequate’, with room for improvement in 51.4% and 45.2% of the two corresponding groups. The main deficiencies in care were recorded evidence of assessment of cervical length (absent in 35% of cases) and failure to screen for bacterial vaginosis in high-risk women.

**CONCLUSIONS:**

Auditing antenatal care for prevention of preterm birth allows identification of suboptimal practice allowing service improvement and potential intervention for preterm birth prevention.

## INTRODUCTION

Preterm birth remains a significant problem, being recognized as one of the most significant contributors to perinatal death in Australia^1^. The most recent report (2021) of the Australian Institute for Health and Welfare (AIHW) reported that 1.6% of births occurred at <32 weeks’ gestation and 6.5% occurred between 32–36 weeks’ gestation^2^. Survivors of preterm birth have a significant risk of ongoing morbidity. One recent population-based series that reported functional developmental assessment of 1204 preterm infants (delivered <28 weeks) made at >18 months age found rates of 23.0%, 8.1% and 4.6% of mild, moderate and severe impairment, respectively^1^. Inpatient costs increase by more than 10% for each week born <39 weeks gestation^3,4^. No recent published economic evaluation of the costs of prematurity in Australia is available, but a recent assessment of the costs of preterm birth in Canada, which has a similar population and healthcare environment, placed immediate costs at >$500 million/year^5^.

Approximately 40% of women in preterm labor present with spontaneously onset of uterine activity where no obvious cause can be defined^6^. There is a growing body of evidence showing that spontaneous preterm birth can, sometimes, be prevented after placing cerclage or using progesterone^7,8^. These data have led some commentators to recommend active screening for prediction of risk and prophylactic intervention to mitigate risk of preterm birth, and this approach is being incorporated into clinical practice^7,8^.

The quality of clinical care can be measured and improved by defining a clear audit cycle^9^. From an obstetric perspective, many outcomes, such as mortality, are infrequent, making it difficult to find a statistically significant difference in the quality of antenatal care provided. This can be addressed either by increasing the population base with national based surveillance schemes or by assessing surrogate ‘near miss’ outcomes, which means in medical practice a potential adverse outcome without actual harm to the patient providing valuable learning opportunities to improve safety and healthcare practices because they highlight weakness in the system^10,11^. The philosophy of ‘near miss’ audit has not been so robustly applied to fetal outcomes, but is well suited to assessment of the quality of obstetric care in relation to preterm birth. Prospective audit related to obstetric management in preterm birth has typically examined clinician’s compliance with protocols for administration of corticosteroids or magnesium sulphate – interventions designed to reduce morbidity of preterm infants^12,13^. There are limited studies in the literature that have assessed the quality of antenatal care in relation to preterm birth^14^. We contend that preterm birth should no longer be considered to be inevitable but is potentially preventable. In this study we have aimed to identify a series of auditable criteria for the assessment of the standard of antenatal care and applied a scoring system that evaluates the quality of obstetric care in relation to the prevention of preterm birth within our own department, allowing service improvement and potential intervention for preterm birth prevention.

## METHODS

### Study design

This was a retrospective, descriptive, longitudinal review of women who had a spontaneous preterm delivery focusing on auditing the quality of antenatal care.

### Setting

The study was conducted at Royal Prince Alfred Hospital, Sydney, Australia, between January 2013 and April 2015. Women were reviewed from the first antenatal visit to delivery.

### Participants

Medical record numbers for women who delivered preterm (24–37 weeks’ gestation) were identified from the labor ward registry. Data related to antenatal care were retrieved from the electronic medical record. Women who had received antenatal care in a different unit, and were transferred for delivery, were excluded from the study. Pregnancies involving multiple fetuses or fetuses with chromosomal or structural anomalies were also excluded.

### Data sources and measurements

Permission to review the clinical notes for these patients was obtained from the hospital ethics committee (RPAH X11-0305) on 25 November 2011. Medical records were reviewed including stored ultrasound images.

### Definitions and diagnostic criteria

*Pregnancy dating*

Pregnancies had been dated by last menstrual period (LMP) with gestational age confirmed by ultrasound. Pregnancies with a crown rump length measurement more than four days different (at the 11–13^+^6 week scan) were redated on the basis of ultrasound findings. For women who did not attend for combined first trimester screening, a biometric difference of more than seven days at the time of the 18–20 week anomaly scan was used as a basis for redating.

*Defining high risk for preterm birth based on history taking*

Women were defined to be in the high-risk group for preterm birth if they have one of the following risk factors: extremes of maternal age (<18 or >35 years), body mass index (BMI <19 or >30 kg/m2), positive maternal history of cardiac or thyroid disease, iron deficiency anemia, diabetes (type I or II) or a history of anxiety and/or depression; a gynecological history of uterine anomaly, recurrent miscarriage or of cervical surgery (Large Loop Excision of Transformation Zone or cone biopsy), and an obstetric history of previous preterm delivery.

*Defining high-risk group based on ultrasonic cervical length*

This scan was performed using a transvaginal approach in our department. Transabdominal assessment was offered for women declining this approach. Closed cervical length ≤25 mm was considered to define a higher level of risk of preterm delivery. Women with a short cervix were offered progesterone or cervical cerclage as preventative interventions and were placed under a higher level of surveillance^8,15^.

*Defining high-risk group based on PaPP-A level during routine combined first trimester screening test*

PaPP-A level at or less than 0.3 MoM was also deemed to increase risk of preterm delivery^16^.

*Defining the appropriate time for identifying bacterial vaginosis*

According to the American College of Obstetrician and Gynecologist, bacterial vaginosis has to be identified as early as the first trimester to allow timely diagnosis and treatment, which may help reduce the risk of preterm birth^17^.

*Defining possible antenatal interventions for high-risk group*

Some women were given progesterone purely on the basis of their clinical history of previous preterm birth, other obstetricians preferred to base any intervention (progesterone or cerclage) on the findings of serial cervical surveillance, with transvaginal sonographic assessment offered at two weekly intervals from 16 to 24 weeks gestation.

*Defining best practice antenatal care*

The definition of ‘best practice’ antenatal care for the prediction of preterm birth and implementation of preventative therapeutic interventions was based on Royal Australian and New Zealand College of Obstetricians and Gynaecologists (RANZCOG) guidelines and published literature^18,19^. This process first involves taking a history at the booking antenatal visit at or before 16 weeks’ gestation to establish risk, based on the risk factors for preterm birth described above. Women deemed to be at low risk of preterm birth on the basis of their clinical history were then routinely screened for asymptomatic bacteriuria (with a mid-stream urine test), normal PaPP-A level as part of routine combined first trimester screening test, and for evidence of cervical shortening by measurement of cervical length at the time of the morphology scan (18–20 weeks’ gestation). While high-risk women, were referred for antenatal care <12 weeks’ gestation where screening for bacterial vaginosis and earlier cervical length screening from 16 weeks is offered, in addition to the usual antenatal care for low-risk women described above.

### Auditable scoring system

An auditable scoring system was created that allowed assessment of quality of antenatal care in relation to adherence to the prescribed protocol for preterm birth^19^. The scoring system for low-risk women evaluated four features: gestational age of booking visit, whether a first trimester combined test was performed, whether cervical length assessment was offered at the anomaly scan, and whether screening for asymptomatic bacteriuria was performed. The scoring system for the high-risk group evaluated two more features: whether earlier screening for cervical length and screening for bacterial vaginosis were offered (graded by gestational age). Thresholds for categorization and cut-off scores were determined by expert consensus within our department, based on the guidelines issued by RANZCOG to reduce preterm birth^19^.

In this audit, women who delivered preterm were first divided into low- and high-risk groups. The scoring systems for low- and high-risk groups are shown in Tables 1 and 2. In the low-risk group, the score will range from 0 to 11, while for the high-risk group from 0 to 15. A good standard of care was defined as one where all, or almost all, criteria were met (scores ≤3 in low-risk, and ≤4 in high-risk groups). An adequate standard of care was defined by scores of 4–8 in low-risk and 5–10 in high-risk groups. Less than adequate care was defined as one where multiple processes were incomplete. This was defined by a score ≥9 in low-risk and ≥11 in high-risk groups.

**Table 1:** The scoring system used to define quality of antenatal care in high-risk pregnancies that subsequently delivered preterm between January 2013 and April 2015

*Actions*	*Scoring points*					
	*5*	*4*	*3*	*2*	*1*	*0*
Booking (weeks)	None	>20			16–20	<16
1st trimester combined test					None	Done
Cervical assessment at 18–20 weeks		None		Transabdominal		Transvaginal
Mid-stream urine					None	Done
At least one cervical assessment between 16 and 23 weeks					None	Done
High vaginal swab (weeks)			>19 or none	16–19	12–16	<12

**Table 2:** The scoring system used to define quality of antenatal care in low-risk pregnancies that subsequently delivered preterm between January 2013 and April 2015

*Actions*	*Scoring points*					
	*5*	*4*	*3*	*2*	*1*	*0*
Booking (weeks)	None	>20			16–20	<16
1st trimester combined test					None	Done
Cervical assessment at 18–20 weeks		None		Transabdominal		Transvaginal
Mid-stream urine					None	Done

### Data analysis

Some measures were categorical – for example a woman either had, or did not have screening for asymptomatic bacteriuria. Others effectively acted as continuous variables – for example the timing of a transvaginal scan to assess cervical length. In order to accommodate both types of variables, we have classified the continues variables into categorical classes. A scoring system was devised that reflected the relative importance of any deviation from ‘best practice’. Descriptive statistical techniques were applied for analysis. The statistical software package SPSS 26.0 (IBM Corp, Armonk, NY, USA) was used for data analysis and statistical significance was declared at α<0.05.

## RESULTS

**Figure 1:**
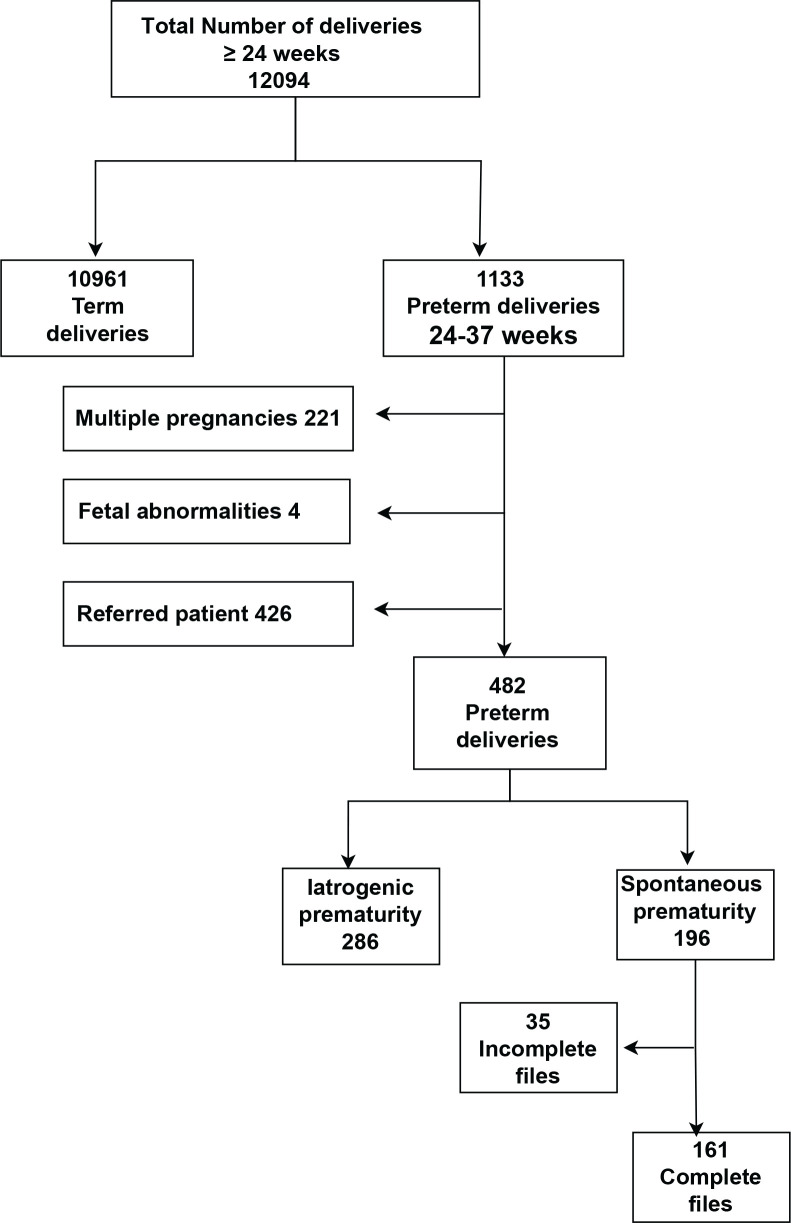
Flow diagram for the participants

A total of 12094 women delivered at ≥24 weeks’ gestation at Royal Prince Alfred Hospital, Sydney, between January 2013 and April 2015. In all, 1133 (9.4%) delivered before 37 weeks’ gestation including 221 with a multiple pregnancy, four with known fetal abnormality, and 426 women either referred for neonatal care or cared for by a private obstetrician. These groups (multiple pregnancy, pregnancies with fetal abnormalities, and referred deliveries) were excluded from subsequent analysis as were women who were delivered for iatrogenic indications (n=286). In the remaining cohort of 196 women who had been booked for antenatal and intrapartum care in our unit, 35 (17.9%) sets of notes were incomplete or inaccessible – so a formal review of performance was not possible. The medical records were available for review in 161 singleton pregnancies impacted by preterm delivery. Figure 1 demonstrates a flow diagram for the participants.

A total of 35 (21.7%) of the pregnancies that resulted in spontaneous preterm delivery were retrospectively identified as been at high-risk at the outset of pregnancy, whilst 126 (78.3%) were low-risk pregnancies. The effectiveness of antenatal management was assessed separately for these two groups (Table 3). Of the high-risk group, 33 (94.3%) had an ‘adequate’ or ‘good’ standard of antenatal care, but there was room for improvement in 18 (51.4%) of these cases. The most common deviation from best practice in this group was failure (17/18 cases) or late (1/18 cases) collection of a high vaginal swab for identification of bacterial vaginosis. In addition, only two of these 18 women had appropriate transvaginal assessment of the cervix: in 7 (39%) cases cervical surveillance was never performed, and in 9 (50%) assessment was limited to a transabdominal approach. Two (6%) of the 35 women defined as being at high risk had suboptimal care. Both were first seen within the hospital >20 weeks’ gestation and neither had had any preventative intervention or cervical surveillance from 16 weeks gestation. One had had cervical assessment at the 20-week anomaly scan – but the method of assessment was not stated on the ultrasound report; 19 (54%) of the 35 high-risk women had had serial cervical surveillance and, although there was no fixed policy for ongoing management, 8 (24%) women had had therapeutic intervention, 3 on the basis of their clinical history and 5 on the basis of the findings of serial cervical surveillance.

**Table 3:** Audited scores of quality of antenatal care for pregnancies that subsequently delivered preterm between January 2013 and April 2015 attributed to low-risk and high-risk groups

*Quality*	*Quality of care score*		*Low-risk cohort (N=126) n (%)*	*High-risk cohort (N=35) n (%)*
	*Low-risk cohort*	*High-risk cohort*		
Good	0–3	0–4	63 (50.0)	15 (42.9)
Adequate	4–8	5–10	57 (45.2)	18 (51.4)
Poor	9–11	11–15	6 (4.7)	2 (5.7)

A total of 120 (95.2%) women in the low-risk group had an ‘adequate’ or ‘good’ standard of antenatal care, but there was room for improvement in 57 (45.2%) of these cases. The major areas identified for improvement included better provision of cervical assessment at the time of the anomaly scan (not performed in 41/57 cases). In 6 (4.7%) women, antenatal care was defined as being ‘poor’. None of these women was seen for antenatal care within the hospital before 20 weeks’ gestation (one presented in labor). None of these women had first trimester screening and 4 had no investigation for asymptomatic bacteriuria. None of them had transvaginal assessment of cervical length at 19 weeks’ gestation.

## DISCUSSION

In our institution, we have defined pathways for the management of pregnant women deemed to be at low or high risk for preterm labor. In this study we used these protocols to develop measures of the quality of antenatal care offered to a cohort of women who subsequently went on to deliver before term.

Given that preterm birth is potentially preventable in some of the cases, identifying opportunities to improve our assessment of risk is important. The scoring system that we used has identified substandard antenatal care among a number of women who delivered preterm. There was room for improvement in care offered to 51.4% of the high-risk cohort and 45.2% in the low-risk cohort. On the other hand, it is important to note that the majority of women received a reasonable standard of care; antenatal management was considered ‘poor’ in only 5.7% and 4.7% of cases correspondingly.

One approach to improving standardization and quality of care is to offer specialist clinics for women deemed high-risk of preterm birth. An audit of such a service offered by the Royal Women’s Hospital showed that over a 10-year period there was a 40–50% reduction in rates of preterm birth in all categories from 24–37 weeks^20^. Whilst a process of structured assessment and management of the risk of preterm birth has led to a reduction in rates of preterm birth amongst non-Aboriginal women, we recognize that additional strategies are required to improve outcomes for aboriginal families^21^. It is also important to recognize that, in our series, 78.3% of preterm deliveries impacted women who would traditionally be identified as being at low risk. The implication of this is that in order to effectively prevent preterm birth, prediction and ongoing management processes need to be in place for all women, not just a high-risk cohort.

In our audit, one of the most significant findings was the lack of a record of cervical length during the 20-week scan. This finding was a consistent feature of a ‘gap’ in care in both low- and high-risk groups; 55 of the total of 161 (34.2%) pregnancies reviewed in this study had no recorded evidence of assessment of cervical length (through either a transvaginal or transabdominal approach). A population based approach involving transvaginal cervical screening has been successfully linked to prophylactic intervention (prescription of progesterone) as a means of reducing the prevalence of preterm birth^7,22^. Whilst there is some controversy about the need for transvaginal cervical assessment cases as routine in all cases, and we recognize that this approach does have resource implications for population based screening, assessment of cervical length is a reportable standard in Australia, so transabdominal cervical length should be reported in all cases as a minimum standard of care^23^. Recognizing this deficiency in care within our population provides opportunities for education of our workforce (GPs, midwives and obstetricians who order the scans, and radiologists who review and report imaging). It is important to reinforce the fact that screening cervical length has been directly associated with therapeutic interventions that reduce rates of preterm delivery^7^. Although, in this study, we did not assess failure to perform cervical assessment in a cohort of women who delivered at term, this will be the subject of future work as a lack of data about cervical length should potentially be considered to be a risk factor for preterm birth. This may impact guidelines such as the UK NHS ‘Saving Babies Lives’ Bundle for preterm birth, which includes a statement on preventative measures and states that healthcare providers should have ‘access to transvaginal cervix scanning’ but does not mandate universal screening^24^.

Only 8 of the 35 high-risk pregnancies were offered progesterone therapeutic intervention aiming to prevent preterm birth during the antenatal period. Similarly, only 54% of this group had serial cervical surveillance despite the fact that in a secondary analysis of a larger study, De Franco et al.^25^ reported that cervical length (<28 mm) was an important discriminator in identifying responders to intervention. There is conflicting evidence supporting the use of progesterone in women who have previously had a preterm birth^22,26^. However, in women with a short cervix, the rate of preterm birth at <32 weeks was significantly lower for those receiving progesterone than placebo (0% vs 29.6%; p=0.014) and neonatal morbidity was reduced^25^.

In our series, 88.5% of high-risk patients were not appropriately screened for bacterial vaginosis. This might be attributed to the lack of evidence-based consensus. Screening and treating bacterial vaginosis is a controversial issue in prediction and prevention of preterm birth. Whilst most guidelines agree that there is little value in screening and treating asymptomatic women at low risk of preterm birth, a variety of approaches to screening/treating high-risk women have been advocated^27,28^. The presence of bacterial vaginosis is associated with an increased risk of preterm delivery and the level of risk is inversely associated with gestational age at diagnosis, but evidence of value of treating women for bacterial vaginosis is weak^29^. Whilst our policy advises that high-risk women should be screened for bacterial vaginosis, it does not recommend a specific intervention strategy. We plan to review this process of investigation and intervention with our senior obstetricians to determine whether the unit should maintain this approach.

The audit tool that we have developed could be altered to reflect local practice, but this study has demonstrated the potential value of formalizing review of the pathway of preterm prevention to be able to identify parts of clinical practice that are not been implemented in an optimal way – allowing service improvement.

### Limitations

Limitations of this study include the retrospective development and application of this scoring system, which has potential for refinement prior to future, prospective review of our clinical service. Future changes to guidelines may affect the scoring system which needs to be updated according to emerging evidence. We note, for example that the UK NHS ‘Saving Babies Lives’ Bundle for prevention of preterm birth stratifies women into three (rather than two) risk groups, does not advocate screening for bacterial vaginosis but advocates assessment of risk of placental insufficiency (and the need for aspirin prophylaxis) and establishing whether women smoke at the onset of pregnancy^24^. Further work, comparing guideline compliance in cohorts of preterm and term deliveries would improve our understanding of the impact of individual components of the current management protocol. We also note that the use of different interventional approaches for women at risk of preterm birth (e.g. use of progesterone versus cervical cerclage) may have introduced some bias to ongoing management.

## CONCLUSIONS

This audit, using a structured tool to review clinical practice, allowed identification of potential deficiencies in the antenatal care of women who delivered preterm. This facilitates service improvement in care designed to predict risk of, and allow prevention of, preterm birth. This process of reflection on current standard of care allows us to improve clinician education related to various aspects of our current policy and provides an opportunity to reassess our management guideline with reference to national and international benchmarks. We anticipate that the quality of care will then be reassessed through the audit cycle.

## Data Availability

The data that support the findings of this study are available from Royal Prince Alfred Hospital. Restrictions apply to the availability of these data, which were used under license for this study. Data are available from the corresponding author, on reasonable request, with the permission of the IRB committee at Royal Prince Alfred Hospital.
